# Simultaneous ManNAc and Neu5Ac Quantification in Human Sera by LC-MS/MS

**DOI:** 10.3390/ijms27020894

**Published:** 2026-01-15

**Authors:** Gerardo N. Guerrero-Flores, Fabio J. Pacheco, Veronica L. Martinez Marignac, Christopher C. Perry, Guangyu Zhang, Martin L. Mayta, Josef Voglmeir, Li Liu, Gary E. Fraser, Fayth M. Butler, Danilo S. Boskovic

**Affiliations:** 1 Centro Interdisciplinario de Investigaciones en Ciencias de la Salud y del Comportamiento (CIICSAC), Facultad de Ciencias de la Salud, Universidad Adventista del Plata, 25 de Mayo 99, Libertador San Martín 3103, Argentina; gerardo.guerrero@uap.edu.ar (G.N.G.-F.); fabio.pacheco@uap.edu.ar (F.J.P.);; 2 Facultad de Ciencias Médicas, Universidad Nacional de Rosario (UNR), Rosario 2000, Argentina; 3 Centro de Investigación Científica y de Transferencia Tecnológica a la Producción (CICYTTP-CONICET), Diamante 3105, Argentina; 4 Division of Biochemistry, Department of Basic Sciences, School of Medicine, Loma Linda University, Loma Linda, CA 92350, USA; 5 Facultad de Ciencias Bioquímicas y Farmacéuticas, Universidad Nacional de Rosario (UNR), Rosario 2000, Argentina; 6 Glycomics and Glycan Bioengineering Research Center (GGBRC), College of Food Science and Technology, Nanjing Agricultural University, Nanjing 210095, China; 7 Center for Nutrition, Healthy Lifestyles and Disease Prevention, School of Public Health, Loma Linda University, Loma Linda, CA 92350, USAfmiles@llu.edu (F.M.B.); 8 Adventist Health Study, Loma Linda University, Loma Linda, CA 92350, USA; 9 Department of Medicine, School of Medicine, Loma Linda University, Loma Linda, CA 92350, USA; 10 Department of Earth and Biological Sciences, School of Medicine, Loma Linda University, Loma Linda, CA 92350, USA

**Keywords:** age, BMI, diet, ethnicity, LC-MS/MS, ManNAc, Neu5Ac, sex

## Abstract

N-Acetyl-D-mannosamine (ManNAc) and N-acetylneuraminic acid (Neu5Ac) are important components of glycosylation, affecting numerous physiologic processes. The effects of age, body mass index (BMI), race, or sex on serum levels of ManNAc and Neu5Ac are poorly understood. However, these associations are of substantial interest. Simultaneous quantification of ManNAc and Neu5Ac, using liquid chromatography tandem mass spectrometry (LC-MS/MS), was developed and validated for human serum samples. This method has high sensitivity, specificity, and reproducibility, with limits of detection as low as 1.02 ng/mL for ManNAc or 1.14 ng/mL for Neu5Ac. A set of 155 serum samples from the Adventist Health Study 2 (AHS-2) cohort was analyzed. Concentrations of conjugated Neu5Ac were 35.1 ± 9.4 µg/mL and 33.0 ± 9.5 µg/mL in black and white participants, respectively. Conjugated and total Neu5Ac levels were significantly higher in women, with *p*-values of 0.029 and 0.026, respectively. The free forms of Neu5Ac were 594 ± 421 ng/mL and 439 ± 168 ng/mL in black and white participants, respectively. Similarly, conjugated and total ManNAc levels were higher in black participants, at 1.81 ± 0.81 µg/mL and 1.90 ± 0.83 µg/mL, compared to 1.32 ± 0.52 µg/mL and 1.41 ± 0.53 µg/mL in white participants (both cases, *p* < 0.001). Free ManNAc was 93.1 ± 36.2 ng/mL in black and 89 ± 20.2 ng/mL in white participants. Subjects with higher BMI tend to have higher free ManNAc (*p* = 0.041). Furthermore, older subjects tend to have higher free (*p* ≤ 0.001) and total (*p* = 0.045) ManNAc. The improved LC-MS/MS quantification method should facilitate further investigations.

## 1. Introduction

*N*-Acetyl-D-mannosamine (ManNAc) is an uncharged monosaccharide produced endogenously by cells [1]. ManNAc, like other acetamido sugars, is a building block commonly incorporated in cell walls, glycolipids, or proteins [2,3,4]. In humans, the cell surface glycans are built mainly using monosaccharides like N-acetylglucosamine (GlcNAc) and N-acetylgalactosamine (GalNAc). In contrast, the presence of ManNAc in cell surface glycans is not well documented [2,5].

**Figure 1:**
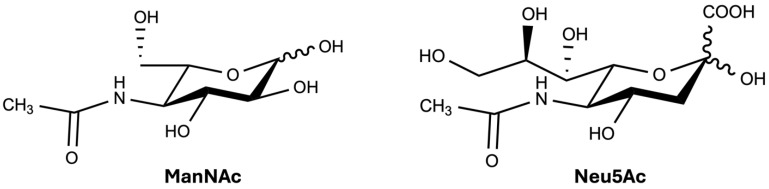
ManNAc and Neu5Ac molecular structures. Prepared with ChemDraw, ver.: 23.1.2.7.

While its role on eukaryotic cell surfaces is not well described, ManNAc is known to be absorbed and metabolized by the cells [5]. Once in the cell, ManNAc can be used to synthesize neuraminic acid, a precursor of the 9-carbon carboxylated monosaccharide, *N*-acetylneuraminic acid (Neu5Ac) (Figure 1), which is the main human form of sialic acid [6,7]. Commonly, Neu5Ac is a conjugated sugar residue found at the termini of oligosaccharide structures in glycoproteins and glycolipids [8,9], and less often in free form [9,10,11,12,13]. Neu5Ac abundance appears to be site-specific and context-dependent in cells and tissues [11,14,15,16,17]. Its synthesis is controlled via enzymatic regulation and dietary intake [12,18].

**Figure 2:**
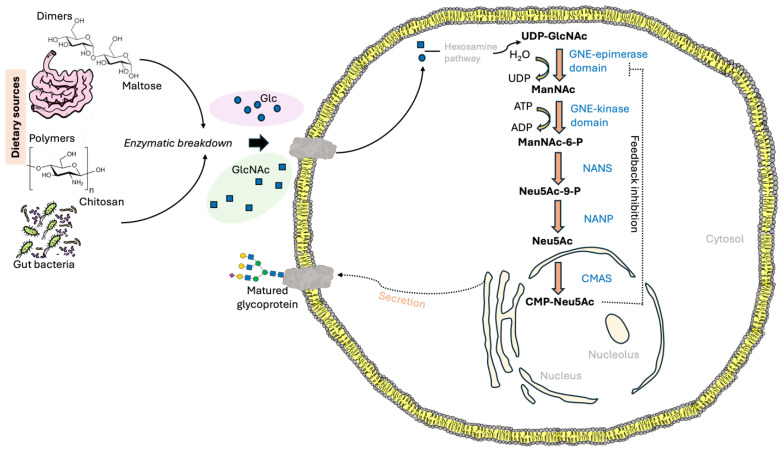
Synthesis of N-acetylneuraminic acid (Neu5Ac) in eukaryotic cells. Dietary carbohydrates reach the gut, where several host and bacterial enzymes break them down into monosaccharides. For instance, enzymes from the host gut, such as maltase, break down disaccharides like maltose into glucose (Glc), while bacterial enzymes break down polymers like chitosan to obtain N-acetylglucosamine (GlcNAc). These monosaccharides enter cells via transporters. Once in the cytoplasm, these sugars participate in the hexosamine pathway. The uridine diphospho-N-acetylglucosamine 2-epimerase/N-acetyl-mannosamine kinase (GNE) enzyme is crucial for regulating Neu5Ac production. It catalyzes two essential steps in the production of Neu5Ac: (a) the conversion of UDP-GlcNAc into ManNAc through epimerization, and (b) the phosphorylation of ManNAc into ManNAc-6-P. Following these steps, the sialic acid synthase (NANS) converts ManNAc-6P to Neu5Ac-9P in a condensation reaction with phosphoenol-pyruvate (PEP). Subsequently, the *N*-acetylneuraminic-acid-phosphatase (NANP) mediates the dephosphorylation to produce Neu5Ac, which is then converted to CMP-Neu5Ac in the nucleus. Both UDP-GlcNAc and CMP-Neu5Ac serve as precursors for N-linked glycan biosynthesis. CMP-Neu5Ac plays a role in inhibiting GNE’s activity via a feedback mechanism.

A series of enzymatic steps in the glycolysis pathway converts glucose into fructose 6-phosphate [19]. Fructose 6-phosphate, in turn, can be converted into uridine diphosphate *N*-acetylglucosamine (UDP-GlcNAc) [20]. At this point, the sialic acid biosynthesis pathway progresses through four cytosolic sequential enzymatic steps [21]. (1) UDP-GlcNAc is epimerized by the *N*-terminal epimerase domain of uridine diphospho-N-acetylglucosamine 2-epimerase/N-acetyl-mannosamine kinase (GNE) to produce *N*-acetylmannosamine (ManNAc) [22]. (2) Using ATP, the kinase domain of GNE phosphorylates ManNAc to produce ManNAc-6P [6,23,24]. (3) Sialic acid synthase catalyzes the condensation reaction of phosphoenolpyruvate (PEP) with ManNAc-6P to form Neu5Ac-9P. (4) Then, Neu5Ac-9P is dephosphorylated by sialic acid phosphatase. In the nucleus, Neu5Ac is activated to form CMP-sialic acid by CMP-Neu5Ac synthetase. The product is carried by the CMP-sialic acid transporter to the Golgi apparatus, where sialyltransferases catalyze the sialylation of various types of glycoconjugates [25] (Figure 2).

A number of factors may influence human levels of ManNAc and Neu5Ac and their physiologic roles, including genetics [15,22], diet [18,26,27,28,29], and age [11,12,30,31]. In this context, the relationship between the levels of particular forms of sialic acids and lifestyle or demographic characteristics remains unclear. For example, it is uncertain how the human diet affects Neu5Ac concentrations in various body compartments. Although conjugated Neu5Ac is found in foods such as red meats, breast milk, bovine milk, eggs, and even in unusual foods such as edible bird’s nests [28,32,33], the impact of such foods on Neu5Ac levels is still unknown. Thus, it seems reasonable to hypothesize that its concentration can be altered by dietary components, though this has not yet been investigated in a cohort study.

Additionally, sialylation levels vary throughout development, growth, and senescence [34,35]. For instance, altered sialylation is seen in aging cells [34]. Consistent with this, in vitro aging models show decreased gene expression of α-2,6-sialyltransferase I, which transfers sialic acid to galactose residues of N-glycans [36]. Such age-associated changes in the terminal modifications of glycans reflect physiologic changes at cellular, tissue, and systemic levels [34]. Furthermore, aberrant Neu5Ac expression in glycoconjugates is characteristic of some pathological states, including cancers. This raises the possibility that certain sialic acids may become promising biomarkers [11,37,38,39,40].

Nonetheless, reliable analytical methods for the quantification of ManNAc and Neu5Ac are essential for the investigation of these monosaccharides in biospecimens [41]. Methods employed for the detection and quantification of ManNAc and Neu5Ac include colorimetry, enzymology, high-performance liquid chromatography (HPLC) coupled with ultraviolet (UV) spectroscopy, Gas Chromatography–Mass Spectrometry (GC-MS), proton nuclear magnetic resonance (H^1^ NMR), and electrochemical methods [42,43,44,45,46,47]. Definitive quantification of analytes, however, can be constrained by methodological limitations on specificity and sensitivity [41]. Some analytic methods for ManNAc or Neu5Ac and their quantification involve derivatization steps prior to liquid chromatography-tandem mass spectrometry (LC-MS/MS) [48,49,50]. However, these derivatization steps lengthen sample preparation and increase assay complexity [51,52].

Additional quantification challenges arise due to low molecular weight, charge, and the presence of multiple hydroxyl groups in ManNAc and Neu5Ac. The chromatographic retention of both compounds from the same sample matrix with a single HPLC column represents a technical challenge [41]. Hence, developing a sensitive, specific method for separating and detecting underivatized ManNAc and Neu5Ac in biospecimens using a single type of HPLC column with a stationary hydrophilic phase is of value.

Thus, the goals of the study were (1) to establish a sensitive method for quantifying free and conjugated forms of ManNAc and Neu5Ac in human serum and (2) to investigate differences in ManNAc or Neu5Ac as they relate to demographic characteristics of a subset of healthy participants in the AHS-2 cohort.

## 2. Results

### 2.1. LC-MS/MS Method Validation for Serum ManNAc and Neu5Ac

**Figure 3:**
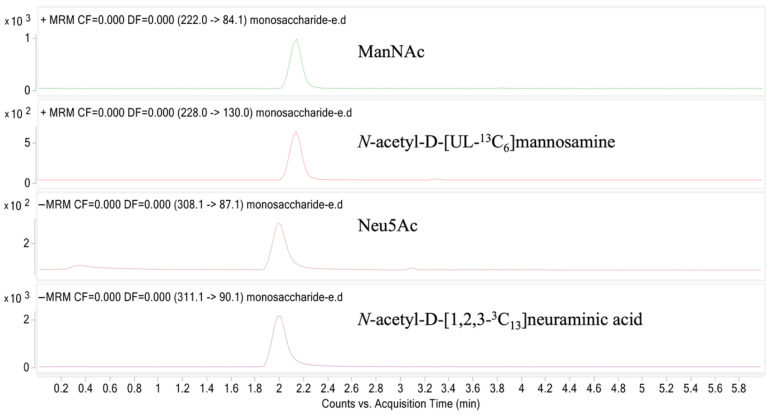
Chromatograms showing signals of ManNAc, *N*-acetyl-D-[UL-^13^C_6_]mannosamine, Neu5Ac, and *N*-acetyl-D-[1,2,3-^13^C_3_]neuraminic acid HPLC peaks on LC-MS/MS, 100 ng/mL each.

The retention times of each compound were monitored to ensure their reproducibility between samples (Table 1, Figure 3). The ManNAc calibration line was constructed using seven standard solutions prepared in ultrapure water: 5, 16, 50, 158, 500, 1581, and 5000 ng/mL. For Neu5Ac, two distinct calibration lines were prepared: (1) Low range: 5, 10, 20, 39, 77, 152, and 300 ng/mL, and (2) High range: 150, 277, 513, 949, 1754, 3244, and 6000 ng/mL.

**Figure 4:**
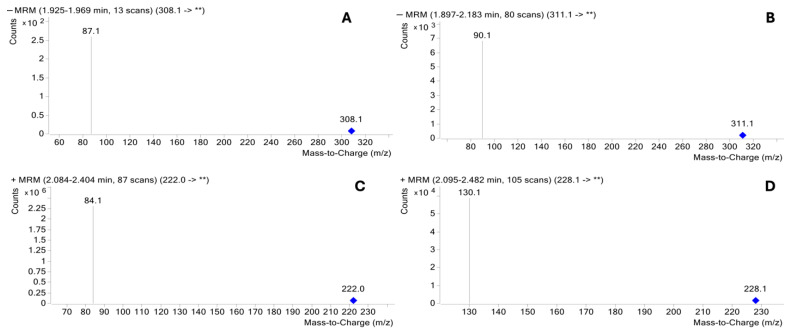
Electrospray ionization mass spectra (ESI-MS) of the daughter scan. (**A**) Neu5Ac at *m*/*z* 87.1, with *m*/*z* 308.1 as the parent ion; (**B**) *N*-acetyl-D-[1,2,3-^13^C_3_]neuraminic acid at *m*/*z* 90.1, with *m*/*z* 311.1 as the parent ion; (**C**) ManNAc at *m*/*z* 84.1, with *m*/*z* 222.0 as the parent ion; (**D**) *N*-acetyl-D-[UL-^13^C_6_]mannosamine at *m*/*z* 130.1, with *m*/*z* 228.1 as the parent ion. ** Designation of the major respective daughter ion peaks in each panel.

The singly charged [M − H]^−^ or [M + H]^+^ parent ions were identified for: Neu5Ac at *m*/*z* 308.0, *N*-acetyl-D-[1,2,3-^13^C_3_]neuraminic acid at *m*/*z* 311.1, ManNAc at *m*/*z* 222 and *N*-acetyl-D-[UL-^13^C_6_]mannosamine at *m*/*z* 228. Selected daughter ions for Neu5Ac, *N*-acetyl-D-[1,2,3-^13^C_3_]neuraminic acid, ManNAc, and *N*-acetyl-D-[UL-^13^C_6_]mannosamine were at *m*/*z* 87.1, 90.1, 84.1, and 130.1, respectively (Figure 4).

**Figure 5:**
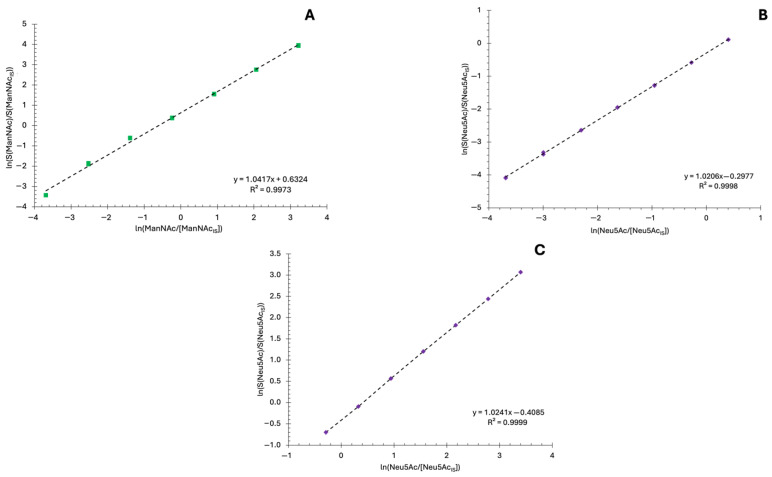
The linearity of the monosaccharides assay. Quantitative calibration curves show the linearity and sensitivity of the LC-MS/MS method for detector signal response (*S*) as a function of the concentrations of free and total ManNAc and Neu5Ac. The lower limits of detection and quantification for Neu5Ac were 1.15 ng/mL and 3.82 ng/mL. For ManNAc, the limits of detection and quantification were 1.02 and 3.41, respectively. Both demonstrate the quantitative sensitivity and robustness of the method. (**A**) Calibration line for ManNAc, where $x=ln\left(\frac{\left[ManNAc\right]}{\left[ManNAc\left(IS\right)\right]}\right)$ and $y=ln\left(\frac{S(ManNAc)}{S(ManNAc\left(IS\right))}\right)$, is expressed as: $ln\left(\frac{S(ManNAc)}{S(ManNAc\left(IS\right))}\right)=1.0417\left(ln\left(\frac{\left[ManNAc\right]}{\left[ManNAc\left(IS\right)\right]}\right)\right)+0.6324$. (**B**) The expression for Neu5Ac is: $ln\left(\frac{S(Neu5Ac)}{S(Neu5Ac\left(IS\right))}\right)=1.0206\left(ln\left(\frac{\left[Neu5Ac\right]}{\left[Neu5Ac\left(IS\right)\right]}\right)\right)-0.2977$. (**C**) The expression for the higher range standard Neu5Ac line is: $ln\left(\frac{S(Neu5Ac)}{S(Neu5Ac\left(IS\right))}\right)=1.0241\left(ln\left(\frac{\left[Neu5Ac\right]}{\left[Neu5Ac\left(IS\right)\right]}\right)\right)-0.4085$. The correlation coefficient (r^2^) for ManNAc and Neu5Ac is > 0.997.

Peak areas for ManNAc or Neu5Ac were corrected by the peak areas of the internal standards *N*-acetyl-D-[UL-^13^C_6_]mannosamine and *N*-acetyl-D-[1,2,3-^13^C_3_]neuraminic acid, respectively. The *r*^2^ values of ManNAc and Neu5Ac were > 0.996, as shown in Figure 5, indicating linearity and sufficient sensitivity for quantification and favorable validation results. Linearity was assessed by calculating the correlation coefficient of calibration lines.

Instrumental limit of detection (*LOD*) and limit of quantification (*LOQ*) were calculated using linearity parameters: standard deviation of blank response (*σ*) and the slope of the standard line (*S*), such that *x_i_* = measurement_i_, *µ* = mean, and *N* = number of measurements [53]:

$$LoD=\frac{3.3\sigma }{S};\;LoQ=\frac{10\sigma }{S};\;\sigma =\sqrt{\frac{\sum {\left(x_{i}-µ\right)}^{2}}{N}}$$

The *LOQ*, the *LOD*, linearity, accuracy (recovery), and precision (intra-assay/inter-assay) were calculated and presented in Table 1 and Table 2. Three quality control solutions were prepared by aqueous dilution of the ManNAc stock solution to obtain 250 (QC_1_), 1000 (QC_2_), and 4000 (QC_3_) ng/mL, and run through the LC-MS/MS to assess the intra-assay and inter-assay variability. Similarly, three control Neu5Ac solutions were prepared, each for low and for high concentration ranges, respectively: 15 (QC_4_), 60 (QC_5_), 240 (QC_6_), or 300 (QC_7_), 1200 (QC_8_), 4800 (QC_9_) ng/mL, for analogous variability assessments. For each of these, the regression lines were derived and presented in Table 1.

### 2.2. ManNAc and Neu5Ac in Serum Samples

**Figure 6:**
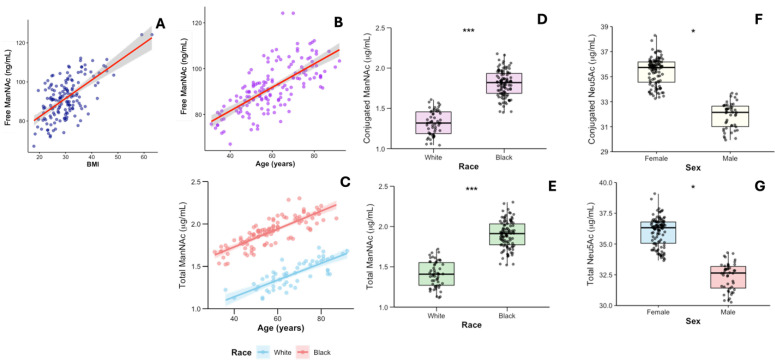
The concentration of ManNAc and Neu5Ac in serum samples (n = 155). (**A**) Association of free ManNAc with BMI, adjusted for age, race, and sex (*p* = 0.040); (**B**) Association between free ManNAc concentration and age, adjusted for BMI, race, and sex (*p* < 0.001); (**C**) Association between total ManNAc and age, adjusted by BMI, race, and sex (*p* = 0.045); (**D**) Association between conjugated ManNAc and race, adjusted for age, BMI, and sex (*p* ≤ 0.001); (**E**) Association of total ManNAc with race, adjusted for age, BMI, and sex (*p* < 0.001); (**F**) Association of conjugated serum Neu5Ac with sex, adjusted for age, BMI, and race (*p* = 0.029); (**G**) Association of total Neu5Ac with sex, adjusted for age, BMI and race (*p* = 0.026). The conjugated form denotes the presence of Neu5Ac as a terminal monosaccharide at the ends of free oligosaccharides, glycolipid glycan chains, and glycoprotein glycan chains. The presence of ManNAc as a conjugate to lipids or protein molecules has not yet been elucidated; nevertheless, similarly to the other acetamido sugars (GlcNAc and GalNAc), this sugar may occupy diverse positions within the glycan structure, thereby modulating its attributes and functionalities. * *p* < 0.05; *** *p* ≤ 0.001. The line shadows, for panels A to C, represent 95% regression confidence intervals.

Sociodemographic information, dietary type, and body mass index (BMI) were obtained from the baseline questionnaire. ManNAc and Neu5Ac were measured and quantified in human serum samples (Table 3). Additionally, the concentrations of these monosaccharides (ng/mL) were compared between black and white cohort members using linear regression analysis and adjusted means (Table 4). Conjugated and total ManNAc concentrations were higher in black compared to white subjects, ß: 0.296, 95% CI: 0.140, 0.440, and ß: 0.285, 95% CI: 0.140, 0.430, both with *p* ≤ 0.001 (Figure 6B, E). Moreover, linear regression reveals a significant direct association between BMI and free ManNAc, ß: 0.006 ng/mL, 95% CI: 0.000, 0.013, *p* = 0.040 (Figure 6C). Similarly, a significant direct age association was observed with free or total ManNAc, ß: 1.070 ng/mL, 95% CI: 1.030, 1.100, and ß: 0.004 ng/mL, 95% CI: 0.0002, 0.0096 (Figure 6A, D), *p* < 0.001 and *p* = 0.045, respectively. Moreover, conjugated and total Neu5Ac are significantly higher in serum samples from women, ß: −0.106 ng/mL, 95% CI: −0.200, −0.010, and ß: −0.107 ng/mL, 95% CI: −0.200, −0.010; *p* = 0.029 and *p* = 0.026, respectively (Figure 6F,G). No significant associations were observed between serum monosaccharide concentrations and dietary types (Table 5).

## 3. Discussion

Simultaneous quantification of free and conjugated forms of ManNAc and Neu5Ac in human sera is presented. The serum samples were obtained from 155 healthy adult participants in the AHS-2 study. As part of this pilot study, significant associations are recognized between the levels of ManNAc or Neu5Ac monosaccharides and participants’ race, BMI, age, or sex.

### 3.1. Methodological Considerations

The use of stable isotope-labeled internal standards for ManNAc or Neu5Ac, *N*-acetyl-D-[UL-^13^C_6_]mannosamine or *N*-acetyl-D-[1,2,3-^13^C_3_]neuraminic acid, respectively, is an analytical strategy described previously [54,55]. This approach relies on the equivalent physicochemical properties of the isotope-labeled standards and the biomarkers tested. Consequently, the labeled standards behave consistently with the biomarkers tested during extraction, instrumental separation, and specific detection [54,56]. Additionally, the use of isotopes provides internal quality control, facilitating the correction for unaccounted measurement variables [57,58] and helping to minimize ion suppression/enhancement effects during the LC-MS/MS analysis [59]. Adding isotope-labeled standards before sample extraction enables a reliable estimation of the concentration of molecules within a sample.

Due to their high polarity and low molecular weight, ManNAc and Neu5Ac can be separated on a HILIC-type stationary phase using mixed aqueous–organic mobile phases, where high-aqueous conditions favor surface-mediated interactions for improved selectivity [60,61,62]. The highly volatile organic mobile phase causes low back pressure due to its reduced viscosity [63]. Additionally, the organic solvent, such as acetonitrile, promotes the formation of smaller droplets due to lower surface tension, improving desolvation efficiency [64,65]. This phenomenon leads to more ions generated within the electrospray source [66] and more ions detected by the mass spectrometer [67,68,69], improving sensitivity.

Separation of polar molecules ManNAc and Neu5Ac, using a single HILIC column, can be challenging, possibly due to low molecular weights, charge, and multiple polar hydroxyl groups. Additionally, at least some of the difficulties may be due to the poor repeatability of the HILIC retention times [60,70,71]. The analysis of ManNAc and Neu5Ac was previously reported using separate columns [41,72]. Separation of ManNAc and Neu5Ac using a single chromatographic procedure, with good retention time repeatability (Table 2), is now worked out.

The presented method meets the high-throughput requirements for both sugars. The technique has the desired accuracy, precision, and sensitivity (Table 1 and Table 2) within wide concentration ranges. Furthermore, the sample preparation process is relatively straightforward. The required sample volume is less than 50 µL, the injection volume is 5 µL, and the total instrument run-time is 6 min, including the column reconditioning after every sample injection. Previously published methods used larger sample volumes and/or longer run times [1,16,41,73,74,75,76,77].

Now, ManNAc and Neu5Ac are measured using a direct and relatively straightforward method, without derivatization steps, using a HILIC column, ensuring good retention time repeatability, good peak shapes, excellent selectivity, and high sensitivity in ESI-MS.

### 3.2. Clinical Significance

Various diseases are associated with unusual concentrations of monosaccharides due to altered glycan synthesis, degradation, or recognition [78]. Studies of such monosaccharides were reported, for example, in cancers [79], cardiovascular diseases [80], GNE myopathy [6], neurodegenerative diseases [81,82], type 2 diabetes [83], and other disorders. Further advances will benefit from a sensitive and specific analytical LC-MS/MS approach.

The employed methods are deemed suitable due to (1) low detection and quantification limits for ManNAc and Neu5Ac, and (2) a relatively straightforward process. Consequently, the described methodology may be implemented routinely for diagnostic or monitoring purposes for individuals with conditions associated with altered ManNAc or Neu5Ac. Furthermore, the current procedures can be adapted to quantify urinary sialic acid levels by incorporating a process to mitigate ion suppression and prevent clogging of the nebulizer needle with salts. This approach could be implemented, for example, to monitor free sialic acid storage disorders. These storage disorders result from either the absence or dysfunction of the transport protein sialin, encoded by the SLC17A5 gene [79,84].

The present study is consistent with a number of previous reports. (a) The concentration of total and conjugated Neu5Ac is higher in women (average age = 60.0) compared to men (average age = 59.6), 36.0 µg/mL vs. 32.4 µg/mL (*p* = 0.026), and 35.4 µg/mL vs. 31.9 µg/mL (*p* = 0.029), respectively. This is consistent with other reports of a significant age-dependent increase in serum sialic acid in postmenopausal women and a notable decrease in men [85]. (b) Total and conjugated ManNAc are higher in black compared to white subjects, 1.90 and 1.41 µg/mL (*p* < 0.001); 1.81 and 1.32 µg/mL, respectively (*p* < 0.001). Race-dependent biological mechanisms may contribute to the higher expression of total and conjugated ManNAc. The presented results agree with studies on ethnic differences in healthy human serum *N*-glycome, reporting the highest abundance of most glycoforms in Ethiopian people [11]. Furthermore, carbohydrates, such as fructose, mannose, and galactose, are metabolized differently in black compared to white populations [86]. (c) Subjects with higher BMI tend to have higher free ManNAc levels (*p* = 0.040). In vivo studies demonstrate that dietary supplementation with ManNAc, as the first committed precursor of Neu5Ac [22,87], prevents obesity and systolic hypertension in mice. This also breaks the association between obesity and hypertension [88]. However, no previous human studies presented a correlation between obesity/BMI and ManNAc levels. (d) Older subjects have higher levels of either free (*p* < 0.001) or total (*p* = 0.045) ManNAc. While the aging process is associated with cognitive dysfunction [89], it is not clear what role is played by ManNAc. Interestingly, an in vivo study reported that ManNAc treatment may have potential therapeutic benefits concerning cognitive dysfunction [90]. In humans, during the aging process, augmentation of ManNAc may be activated for compensatory synthesis of Neu5Ac and the polysialic acid chains. These carbohydrates are critical constituents of brain gangliosides, which modulate neural cell adhesion molecules. The association of ManNAc and Neu5Ac with brain functions in epidemiological studies, however, remains to be more fully explored.

The aging process involves a remodeling of the immune system. Such remodeling is associated with chronic low-grade inflammation (inflammaging) [91,92]. Cellular processes are closely regulated. Examples of such regulated processes include the control and maintenance of chromosomes, transcriptional processes, nuclear transport, cytoskeletal structure, autophagy, extracellular signaling, and extracellular matrix [93,94]. Increased ManNAc synthesis, during the aging process, may occur to facilitate Neu5Ac synthesis and its incorporation into cell membrane glycoconjugates. This can be viewed as a compensatory physiological response to mitigate or counteract inflammation [29]. Interestingly, the expression of α-2,6-sialyltransferase I (*ST6Gal I*), which transfers sialic acid to galactose residues of N-glycans, decreases in aging cells [36]. This explains lower Neu5Ac, despite increased cellular synthesis of ManNAc. While it is unclear how gender affects the expression of ManNAc or Neu5Ac, it is recognized that the serum *N*-glycome profile is age and sex dependent [95].

To date, the conjugated form of ManNAc in human sera has not been reported, and detailed pathways incorporating all intermediates in the synthesis of 9-carbon sugars remain unclear [96]. ManNAc is a neutral molecule, which may help in its passage through membranes. In vitro studies indicate that ManNAc enters the intracellular space either by passive diffusion or via an unknown membrane transporter [24,97]. Additionally, significant levels of GlcNAc and ManNAc were reported among the products of fermentation, implying that they can also be exported by yet unidentified transporters [96,98].

The hypothesis of a compensatory mechanism involving sialic acids as mitigators of inflammatory processes remains to be confirmed. The activation of various compensatory anti-inflammatory or anti-inflammaging responses is expected to prevent excessive tissue damage [99]. Adopting a healthy lifestyle in later life can extend lifespan, even for individuals with higher genetic risks [100,101]. In such a context, the higher concentrations of ManNAc and Neu5Ac, rather than causing specific diseases, may represent physiologic attempts to compensate for inflammatory processes occurring in a variety of conditions.

The strengths of this study include the use of samples from a well-characterized epidemiological population. Additionally, the small volumes of human serum samples, the incorporation of internal standards, and quality control procedures optimized the use of samples and ensured accurate and consistent measurement of ManNAc and Neu5Ac. Furthermore, these monosaccharides were quantitatively measured using a direct and relatively straightforward method, without derivatization steps, with analyte separation on the same column. Although race and BMI are shown to be key factors determining the concentration of ManNAc and Neu5Ac, physical activity and a variety of other variables could help to provide an improved understanding.

There are several limitations of this study. First, while focusing on the quantification of the conjugated ManNAc and Neu5Ac, no effort was made to characterize and quantify the specific forms of conjugation involved. This is likely of particular interest since conjugated ManNAc in human serum was not previously reported. Second, it might be suggested that some Neu5Ac could hypothetically be converted to ManNAc during the mild hydrolysis step. It is mechanistically difficult to see this as a likely explanation for the quantity of conjugated ManNAc reported here. If such were the case, however, then one would expect a relatively strong correlation between total ManNAc and total Neu5Ac. Instead, the opposite is observed. The strongest, albeit weak, correlation is seen between free ManNAc and free Neu5Ac (Supplemental Figures S1–S4, Supplemental Table S1), quantifications which involved no procedural hydrolysis. This weak association, however, can be readily rationalized by a dependence of the Neu5Ac on ManNAc as its early precursor in the synthetic pathway. Third, no other columns were used to compare the yield with the HILIC column for the separation of both ManNAc and Neu5Ac. Fourth, limited external validity in this study is implied, given the modest sample size and population. Fifth, further exploration of ethnic effects, beyond the distinction between black and white, may provide additional new insights.

## 4. Materials and Methods

### 4.1. Reagents and Materials

The Accucore HILIC column (150 mm × 4.6 mm, 2.6 μm particles) was obtained from Thermo Scientific (Waltham, MA, USA) and connected to an Accucore HILIC precolumn (10 mm × 4.6 mm, 2.6 μm). Ammonium formate, trifluoroacetic acid (TFA), and acetone were obtained from Fisher Scientific (Carlsbad, CA, USA). HPLC-grade acetonitrile was obtained from Avantor (Radnor, PA, USA). *N*-acetyl-D-[1,2,3-^13^C_3_]neuraminic acid, Neu5Ac, and methanol were purchased from Sigma-Aldrich (St. Louis, MI, USA), while ManNAc and *N*-acetyl-D-[UL-^13^C_6_]mannosamine were obtained from Omicron Biochemicals, Inc. (South Bend, IN, USA). Milli-Q water (GenPure Pro ultrapure water system with UV-photo-oxidation and TOC monitor, by Thermo Scientific Inc., Waltham, MA, USA) was used throughout the study protocol.

The instrumentation included an Agilent 1200 HPLC coupled with a triple quadrupole mass spectrometer 6410 Agilent Technologies detector (Santa Clara, CA, USA). Data processing was performed using Agilent Mass Hunter Software, version B.08.00.

### 4.2. Human Blood Sera

The present study analyzed ManNAc and Neu5Ac concentrations in 155 human serum samples obtained from participants enrolled in the Adventist Health Study 2 (AHS-2) cohort. Established between 2002 and 2007, the AHS-2 cohort comprises over 96,000 Seventh Day Adventists in the USA and Canada. Several studies were conducted with this cohort linking lifestyle choices, particularly diet, to health outcomes [102]. At the commencement of this study, participants completed a comprehensive food frequency questionnaire. Baseline sociodemographic data and body mass index (BMI) were derived from the completed questionnaires.

#### Ethical Considerations

This study was reviewed and approved by the Institutional Review Board of Loma Linda University (IRB #48134, #5190039). The procedures associated with this project were managed following the international ethical standards recommended by the Helsinki Protocol for human research, and informed consent was obtained from all participants.

### 4.3. ManNAc and Neu5Ac Analysis from Serum Samples

Blood samples from healthy subjects were drawn by venipuncture, clotted, and centrifuged. The sera were separated and frozen in liquid nitrogen until further processing for analysis [102]. The proposed method for simultaneous quantitative determination of ManNAc and Neu5Ac in human sera, with a single column using LC-MS/MS, was established after adaptations of previously published methods [29,41]. Briefly, the standard samples were prepared by dilution in ultrapure water. *N*-acetyl-D-[uniformly labeled (UL)-^13^C_6_]mannosamine and *N*-acetyl-D-[1,2,3-^13^C_3_]neuraminic acid were used as internal isotope labeled standards (IS) for ManNAc and Neu5Ac, respectively [72]. Utilization of these internal standards (ISs) throughout sample pre-treatment, chromatographic separation, and mass spectrometry detection enhances the accuracy and precision of quantitation by enabling the corrections for unaccounted measurement variables. Separately, positive controls containing *N*-acetyl-D-[UL-^13^C_6_]mannosamine or *N*-acetyl-D-[1,2,3-^13^C_3_]neuraminic acid (200 ng/mL) in ultrapure water were analyzed during and after each batch analysis for consistency evaluation. Ten samples were analyzed per batch.

### 4.4. Extraction of ManNAc and Neu5Ac from Serum Samples

Before processing human sera, each sample was thawed at room temperature, cooled on ice, and vortexed for 30 sec. Four serum aliquots per subject were used for the extraction of the monosaccharides: (1) ManNAc total, (2) ManNAc free, (3) Neu5Ac total, and (4) Neu5Ac free.

**Figure 7:**
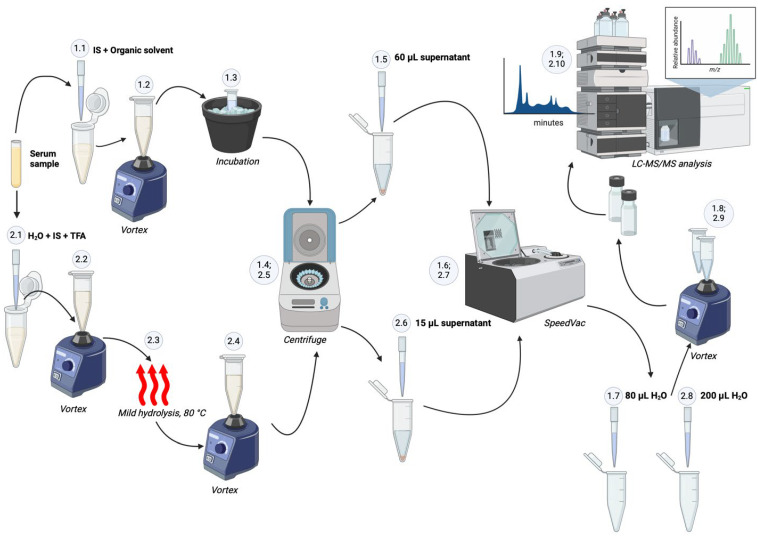
Extraction of free or total (free + conjugated) ManNAc and Neu5Ac from serum samples. Starting with the serum sample, for the analysis of free ManNAc and Neu5Ac, the extraction was as follows: (1.1) A 40 µL aliquot of serum is mixed with 10.6 µL of 5 µg/mL IS stock solution (either *N*-acetyl-D-[1,2,3-^13^C_3_]neuraminic acid or *N*-acetyl-D-[UL-^13^C_6_]mannosamine), to which a cold mixture (149.4 µL, −20 °C) of CH_3_OH:CH_3_CN:CH_3_COCH_3_ (1:1:1) is added; (1.2) the samples are vortexed for 30 s; (1.3) incubated on ice for 30 min; (1.4) followed by centrifugation at 15,000× *g* for 7 min; (1.5) from which 60 μL of supernatant is taken; (1.6) and concentrated; (1.7) the dried free fractions are then reconstituted in 80 μL of pure water; (1.8) vortexed; and (1.9) analyzed using the LC-MS/MS procedure. The extraction of total ManNAc and Neu5Ac from serum samples is as follows: (2.1) 4 µL of serum samples are mixed with 4.7 µL of water, to which 21.3 µL of 5 µg/mL IS stock solution (either *N*-acetyl-D-[1,2,3-^13^C_3_]neuraminic acid or *N*-acetyl-D-[UL-^13^C_6_]mannosamine) and 10 µL of 1 M TFA (250 mM final) are added; (2.2) the mixture is thoroughly vortexed for 30 s; (2.3) incubated at 80 °C with agitation at 400 rpm for 1 h; (2.4) vortexed for 30 s; (2.5) centrifuged at 15,000× *g* for 5 min; (2.6) followed by separation of 15 µL of supernatant; (2.7) concentration; (2.8) reconstitution in 200 µL of pure water; (2.9) vortexing for 30 s; and (2.10) analysis in the LC-MS/MS system. Figure created in BioRender (Lansangan, C. (2025) https://BioRender.com/j69c027).

The total (free + conjugated) ManNAc or Neu5Ac was evaluated as follows. After vortexing, 4 µL of serum was combined with 4.7 µL of pure water, 21.3 µL of 5 µg/mL IS *N*-acetyl-D-[UL-^13^C_6_]mannosamine or *N*-acetyl-D-[1,2,3-^13^C_3_]neuraminic acid, and 10 µL of 1 M TFA solution (250 mM final). The mixture was thoroughly vortexed for 30 s, and then incubated at 80 °C, with agitation at 400 rpm using a Vortemp 56 Labnet shaking incubator (Thermo Fisher Scientific, Waltham, MA, USA) for 1 h, to release ManNAc and Neu5Ac from lipids or proteins. After mild acid hydrolysis, the sample was centrifuged (15,000× *g*, 5 min). Of the supernatant, 15 µL was removed and concentrated using SpeedVac (Savant SC 110A, Thermo Electron Corporation, Waltham, MA, USA). The dried sample was then reconstituted in 200 μL of water, vortexed, and analyzed by LC-MS/MS (Figure 7).

The free ManNAc or Neu5Ac was evaluated as follows. A 40 µL aliquot of serum was mixed with 10.6 µL of 5 µg/mL IS (either *N*-acetyl-D-[UL-^13^C_6_]mannosamine or *N*-acetyl-D-[1,2,3-^13^C_3_]neuraminic acid). A cold mixture (149.4 µL, −20 °C) of CH_3_OH:CH_3_CN:CH_3_COCH_3_ (1:1:1) was added, followed by vortexing for 30 s and incubation on ice for 30 min. The sample was centrifuged at 15,000× *g* for 7 min, followed by drying of 60 μL of supernatant in the SpeedVac. The dried free fraction was reconstituted in 80 μL of water, vortexed, and analyzed by LC-MS/MS (Figure 7). The level of conjugated forms (ManNAc or Neu5Ac) was indirectly determined by subtracting the free sugar concentration from the total amount measured in each sample [103,104].

### 4.5. LC-MS/MS Analysis of ManNAc and Neu5Ac

The LC-MS/MS assay, developed in compliance with the Food and Drug Administration (FDA) Bioanalytical Method Guidelines [105], was applied to a set of serum samples from the AHS-2. HPLC provided adequate separation of ManNAc and Neu5Ac. The mobile phase used in the chromatographic separation included a binary gradient. Mobile phase A contained 10% HCOONH_4_, 0.01% HCOOH in water, while mobile phase B was 10% HCOONH_4_, 0.01% HCOOH in CH_3_CN. The gradient program, with a flow rate of 1 mL/min, was as follows: 0–2 min, 2% B; 2–5 min, gradient increased to 90% B; 5–6 min, gradient returned to 2% B (Table 6). This implies that the HILIC column is not used in the customary manner of HILIC chromatography. Rather, the conditions were chosen to optimize the retention of ManNAc and Neu5Ac, while minimizing it for numerous other hydrophilic serum components. The chromatography eluate was provided to the mass spectrometer only during the time range of 1.6–2.4 min, which includes retention times of ManNAc and Neu5Ac. Otherwise, both head and tail of the chromatographic run were diverted to waste by the 2-position/6-port switching valve. The column temperature was maintained at a constant 30 °C. The injection volume for all samples, QCs, and calibrators was set to 5 μL. The HPLC autosampler was temperature-controlled at 4 °C.

The electrospray ionization-mass spectrometry (ESI-MS) data were combined with tandem mass spectrometry in negative or positive ion mode [41,72]. Quantitation by multiple reaction monitoring (MRM) analysis was performed in the negative ion mode for Neu5Ac and *N*-acetyl-D-[1,2,3-^13^C_3_]neuraminic acid, while the analysis for ManNAc and *N*-acetyl-D-[UL-^13^C_6_]mannosamine was carried out in the positive ion mode (Table 7). The ion spray voltage was operated at −4 KV, and the source temperature was 300 °C. The nebulizer was set at 35 psi, and the gas flow setting was 11 L/min. The ESI-MS of parent and daughter ions, for Neu5Ac, *N*-acetyl-D-[1,2,3-^13^C_3_]neuraminic acid, ManNAc, and *N*-acetyl-D-[UL-^13^C_6_]mannosamine were chosen because of their relative abundances [29,41,72] (Figure 4).

### 4.6. Statistical Analysis

To investigate potential associations between the demographic characteristics of these participants and free, conjugated, or total ManNAc or Neu5Ac, non-parametric statistical methods were employed due to non-normal distributions, as assessed by the Shapiro–Wilk normality test. Mann–Whitney U tests were performed for continuous variables. The chi-square test was used to compare categorical variables, specifically diet, sex, and race. To explore the relationship between race and the free, conjugated, and total concentrations of ManNAc and Neu5Ac, multiple linear regression models were generated after first log-transforming the dependent variables. Models were generated with various independent variables of interest, including diet, BMI, and demographic factors like gender or race. Their associations were explored with serum levels of ManNAc and Neu5Ac. Separate models were generated for each outcome variable: free ManNAc, conjugated ManNAc, and total ManNAc; free Neu5Ac, conjugated Neu5Ac, and total Neu5Ac. The models were subsequently checked for normality using Q-Q plots and the Shapiro–Wilk test for the residuals. Where applicable, back-transformations were performed to interpret the coefficients in the original scale. Adjusted R-squared values and F-statistics were reported to assess model fitness. Statistical significance was determined at *p* < 0.05. All analyses were conducted in RStudio 2024.09.0+375 and R version 4.4.12.4.

## 5. Conclusions

A relatively straightforward HPLC-MS/MS method is presented for the simultaneous separation, detection, and quantification of ManNAc and Neu5Ac from human serum. Notable differences in these monosaccharides are associated with age, BMI, sex, and race of participants in the AHS-2 cohort. Free ManNAc is associated with BMI and age. Additionally, conjugated and total ManNAc are higher in black compared to white subjects. On the other hand, conjugated and total Neu5Ac are significantly higher in women compared to men. Improved ManNAc and Neu5Ac quantification could facilitate studies of the roles of carbohydrates in various disorders, therapeutic approaches, and advancements in personalized medicine. In this regard, metabolic studies, as part of comprehensive multi-omics analyses, can be helpful in identifying pivotal genes and elucidating potential metabolic pathways. Such tools may point out the potential exporters of ManNAc and Neu5Ac.

## Figures and Tables

**Table 1:** Assay Validation of ManNAc and Neu5Ac (n = 21).

Compound	Retention Time (min)	Calibration Curve	r^2^	Concentration Range (ng/mL)	LOD (ng/mL)	LOQ (ng/mL)
ManNAc ^1^	2.21 ± 0.01	y = 1.0417x − 0.6324	0.997	5–5000	1.02	3.41
Neu5Ac_low_ ^2^	2.00 ± 0.01	y = 1.0206x − 0.2977	0.999	5–300	1.15	3.83
Neu5Ac_high_ ^2^		y = 1.0241x − 0.4085	0.999	150–6000		

LOD, limit of detection; LOQ, limit of quantification. ^1^ $x=ln\left(\frac{\left[ManNAc\right]}{\left[ManNAc\left(IS\right)\right]}\right)$, $y=ln\left(\frac{S(ManNAc)}{S(ManNAc\left(IS\right))}\right).$^2^ $x=ln\left(\frac{\left[Neu5Ac\right]}{\left[Neu5Ac\left(IS\right)\right]}\right)$, $y=ln\left(\frac{S(Neu5Ac)}{S(Neu5Ac\left(IS\right))}\right).$

**Table 2:** Intra and Inter-assay Precision for Measurement of ManNAc and Neu5Ac.

Compound	QC	Conc. (ng/mL)	Intra-Assay	Precision (CV %)	Accuracy (%)	Conc. (ng/mL)	Inter-Assay	Precision (CV %)	Accuracy (%)
			Mean ± SD (ng/mL)				Mean ± SD (ng/mL)		
ManNAc	Low	250	242.8 ± 2.3	0.93	97.1	250	205.3 ± 2.1	1.02	82.1
	Mid	1000	952.0 ± 4.0	0.42	95.2	1000	810.2 ± 3.7	0.45	81.0
	High	4000	3975.3 ± 3.7	0.09	99.9	4000	3436.7 ± 3.0	0.09	84.7
Neu5Ac_low_	Low	15	14.5 ± 0.1	0.64	96.6	15	15.3 ± 0.2	1.07	102.0
	Mid	60	59.2 ± 0.3	0.46	99.4	60	61.2 ± 0.9	1.44	102.0
	High	240	240.5 ± 0.4	0.16	100.2	240	242.4 ± 3.7	1.53	101.0
Neu5Ac_high_	Low	300	263.2 ± 0.8	0.31	87.7	300	273.2 ± 3.5	1.28	91.1
	Mid	1200	1066.5 ± 1.9	0.18	88.9	1200	1064.0 ± 0.9	0.09	88.7
	High	4800	4495.7 ± 2.4	0.05	93.7	4800	4313.5 ± 0.2	0.00	89.9

CV, coefficient of variation; QC, quality control.

**Table 3:** Demographic Characteristics of Study Population by Race ^1^.

	Black	White	*p*-Value
Participants			
Age (years)	56.2 (13.0)	67.0 (12.7)	<0.001
Female, n (%)	74 (72.5)	35 (66.0)	
Male, n (%)	28 (27.5)	18 (34.0)	0.512
BMI (Kg/m^2^)	31.1 (7.2)	28.4 (6.2)	0.025
Neu5Ac_Free_ (ng/mL)	594 (421.0)	439 (168.0)	0.028
Neu5Ac_Conj_ (µg/mL)	35.1 (9.4)	33.0 (9.5)	0.110
Neu5Ac_Total_ (µg/mL)	35.7 (9.5)	33.4 (9.6)	0.096
ManNAc_Free_ (ng/mL)	93.1 (36.2)	89 (20.2)	0.947
ManNAc_Conj_ (µg/mL)	1.81 (0.81)	1.32 (0.52)	<0.001
ManNAc_Total_ (µg/mL)	1.90 (0.83)	1.41 (0.53)	<0.001
Diet			
Vegan, n (%)	22 (21.6)	27 (50.9)	0.001 *
Lacto-ovo, n (%)	40 (39.2)	15 (28.3)	
Non-veg, n (%)	40 (39.2)	11 (20.8)	

If not specified, values are presented as mean (SD). Total samples, N_T_ = 155. ^1^ *p*-values computed using Mann–Whitney U test for continuous variables and chi-square test for categorical variables. * There is a statistically significant difference in diet type distribution between Black and White participants.

**Table 4:** Association of BMI, Sex, Race, and Age with ManNAc and Neu5Ac. Beta-coefficients Obtained from Linear Regression Represent a Change in ManNAc and Neu5Ac Concentration According to the Predicted Variable. All the Predictor Variables were Adjusted by Diet Types.

Compound	BMI		Sex		Race		Age *	
	β Coefficient (95% CI)	*p*	β Coefficient (95% CI)	*p*	β Coefficient (95% CI)	*p*	β Coefficient (95% CI)	*p*
Neu5Ac_free_	0.006 (−0.004, 0.017)	0.242	−0.073 (−0.230, 0.080)	0.365	0.163 (−0.010, 0.330)	0.069	0.985 (0.930, 1.040)	0.523
Neu5Ac_conj_	0.002 (−0.004, 0.009)	0.448	−0.106 (−0.200, −0.010)	0.029	0.053 (−0.050, 0.150)	0.317	−0.001 (−0.004, 0.002)	0.597
Neu5Ac_total_	0.002 (−0.003, 0.009)	0.422	−0.107 (−0.200, −0.010)	0.026	0.055 (−0.040, 0.160)	0.290	−0.001 (−0.004, 0.002)	0.580
ManNAc_free_	0.006 (0.000, 0.013)	0.040	−0.037 (−0.131, 0.050)	0.435	0.071 (−0.030, 0.170)	0.178	1.070 (1.030, 1.100)	<0.001
ManNAc_conj_	0.008 (−0.008, 0.010)	0.859	−0.103 (−0.240, 0.030)	0.142	0.296 (0.140, 0.440)	<0.001	0.004 (−0.0001, 0.009)	0.057
ManNAc_total_	0.001 (−0.007, 0.010)	0.793	0.001 (−0.007, 0.010)	0.793	0.285 (0.140, 0.430)	<0.001	0.004 (0.0002, 0.0096)	0.045

* Beta-coefficients for age and CIs were converted to a 10-year age metric. Thus, e.g., free ManNAc increased by 7% over a decade. (Percentage increase = 1.07 − 1) × 100. BMI, body mass index. Sex, men compared to women. Race, black compared to white. CI bounds are presented to 4 decimal places when necessary to reflect values near 0.

**Table 5:** Adjusted Marginal Means (95% CI) of ManNAc and Neu5Ac (free conjugated and total) by Dietary Types (vegan, lacto-ovo, and non-vegetarian) from Linear Regression Models, Adjusted for Age, BMI, Gender, and Race.

Compound	Dietary Type Comparison	Ratio, ng/mL (95% CI)	*p*
Neu5Ac_free_	Vegan vs. Non-vegetarian	1.018 (0.80, 1.28)	0.98
	Vegan vs. Lacto-ovo	0.999 (0.79, 1.24)	0.99
	Non-vegetarian vs. Lacto-ovo	1.019 (0.82, 1.26)	0.97
Neu5Ac_conj_	Vegan vs. Non-vegetarian	−0.034 (−0.14, 0.07)	0.54
	Vegan vs. Lacto-ovo	0.011 (−0.09, 0.11)	0.83
	Non-vegetarian vs. Lacto-ovo	0.046 (−0.07, 0.16)	0.43
Neu5Ac_total_	Vegan vs. Non-vegetarian	−0.033 (−0.14, 0.07)	0.53
	Vegan vs. Lacto-ovo	0.011 (−0.09, 0.11)	0.82
	Non-vegetarian vs. Lacto-ovo	0.045 (−0.07, 0.16)	0.44
ManNAc_free_	Vegan vs. Non-vegetarian	1.042 (0.90, 1.19)	0.75
	Vegan vs. Lacto-ovo	1.005 (0.88, 1.14)	0.99
	Non-vegetarian vs. Lacto-ovo	1.036 (0.91, 1.17)	0.77
ManNAc_conj_	Vegan vs. Non-vegetarian	0.133 (−0.02, 0.29)	0.10
	Vegan vs. Lacto-ovo	0.010 (−0.14, 0.16)	0.89
	Non-vegetarian vs. Lacto-ovo	−0.123 (−0.29, 0.04)	0.15
ManNAc_total_	Vegan vs. Non-vegetarian	0.123 (−0.03, 0.27)	0.11
	Vegan vs. Lacto-ovo	0.011 (−0.13, 0.15)	0.87
	Non-vegetarian vs. Lacto-ovo	−0.112 (−0.27, 0.04)	0.17

CI, confidence interval; BMI, body mass index.

**Table 6:** Gradient program of the mobile phase for HPLC separation.

Time (min)	Mobile Phase		Flow Rate (mL/min)
	A (%) ^a^	B (%) ^b^	
0.0	98.0	2.0	1.0
1.0	98.0	2.0	1.0
2.0	10.0	90.0	1.0
4.0	10.0	90.0	1.0
5.0	98.0	2.0	1.0
6.0	98.0	2.0	1.0

^a^: 10 mM ammonium formate in water; ^b^: 10 mM ammonium formate in acetonitrile (ACN).

**Table 7:** Multiple Reaction Monitoring Transitions for ManNAc and Neu5Ac.

Compound	Precursor Ion (*m*/*z*)		Product Ion (*m*/*z*)	CE (eV)
ManNAc	222	→	84.1	22
*N*-acetyl-D-[UL-^13^C_6_]mannosamine	228	→	130	8
Neu5Ac	308.1	→	87.1	18
*N*-acetyl-D-[1,2,3-^13^C_3_]neuraminic acid	311.1	→	90.1	12

CE = collision energy; eV = electron volts; *m*/*z* = mass to charge ratio.

## Data Availability

Correspondence and material requests should be addressed to the corresponding author.

## Supplementary Materials

The following supporting information can be downloaded at: https://www.mdpi.com/article/10.3390/ijms27020894/s1.
