# Supplementary material for: Simultaneous ManNAc and Neu5Ac Quantification in Human Sera by LC-MS/MS
Source: Int J Mol Sci. 2026 Jan 15;27(2):894. doi: 10.3390/ijms27020894 (PMC12841319; doi:10.3390/ijms27020894)
Supplement: Supplementary file 1 [file ijms-27-00894-s001.zip › ijms-4030742-supplementary.pdf]

Supplementary Information

Simultaneous ManNAc and Neu5Ac Quantification by LC-MS/MS using Adventist Health Study 2 Participants' Sera

Gerardo N. Guerrero-Flores[1,2], Fabio J. Pacheco[1], Veronica L. Martinez-Marignac[3], Christopher C. Perry[4], Guangyu Zhang[4], Martin L. Mayta[1,5], Josef Voglmeir[6], Li Liu[6], Gary E. Fraser[7,8,9], Fayth M. Butler[7,8] and Danilo S. Boskovic[4,10,*]

[1]Centro Interdisciplinario de Investigaciones en Ciencias de la Salud y del Comportamiento (CIICSAC), Facultad de Ciencias de la Salud, Universidad Adventista del Plata, 25 de Mayo 99, 3103, Libertador San Martín, Entre Ríos, Argentina

[2]Facultad de Ciencias Médicas, Universidad Nacional de Rosario (UNR), Rosario, 2000 Argentina

[3] Centro de Investigación Científica y de Transferencia Tecnológica a la Producción (CICYTTP-CONICET), Diamante, 3105 Argentina

[4]Division of Biochemistry, Department of Basic Sciences, School of Medicine, Loma Linda University, Loma Linda, CA, 92350, USA

[5]Facultad de Ciencias Bioquímicas y Farmacéuticas, Universidad Nacional de Rosario (UNR), 2000 Rosario, Argentina

[6]Glycomics and Glycan Bioengineering Research Center (GGBRC), College of Food Science and Technology, Nanjing Agricultural University, Nanjing, People's Republic of China

[7]Center for Nutrition, Healthy Lifestyles and Disease Prevention, School of Public Health, Loma Linda University, Loma Linda, CA, 92350, USA

[8]Adventist Health Study, Loma Linda University, Loma Linda, CA 92350, USA

[9]Department of Medicine, School of Medicine, Loma Linda University, Loma Linda, CA 92350, USA

[10]Department of Earth and Biological Sciences, School of Medicine, Loma Linda University, Loma Linda, CA 92350, USA

* Corresponding author: dboskovic@llu.edu

ManNAc is an early precursor in the pathway of Neu5Ac synthesis (Figure 2). Consequently, it is not unreasonable to expect some association between the concentrations of ManNAc and Neu5Ac. Indeed, consistent with this expectation, Figure S1 presents a weak but positive association between the free forms of ManNAc and Neu5Ac.

Any suggestion that some ManNAc could hypothetically be produced via hydrolysis of Neu5Ac is not easy to rationalize mechanistically. If such a process were to occur, then one would expect that the correlations between conjugated or total ManNAc and Neu5Ac would be higher than that observed between the free forms. Instead, what is observed is that the correlation between conjugated forms is actually weaker than that observed between the free forms, which were not subjected to a hydrolysis step (Figures S2, S3, Table S1). Moreover, since total ManNAc and Neu5Ac levels include the respective free forms, the correlation between the total forms is slightly higher than that for the conjugated forms, but less than that between the free forms.

In conclusion, the measured levels of conjugated ManNAc cannot be explained to represent products of Neu5Ac hydrolysis.

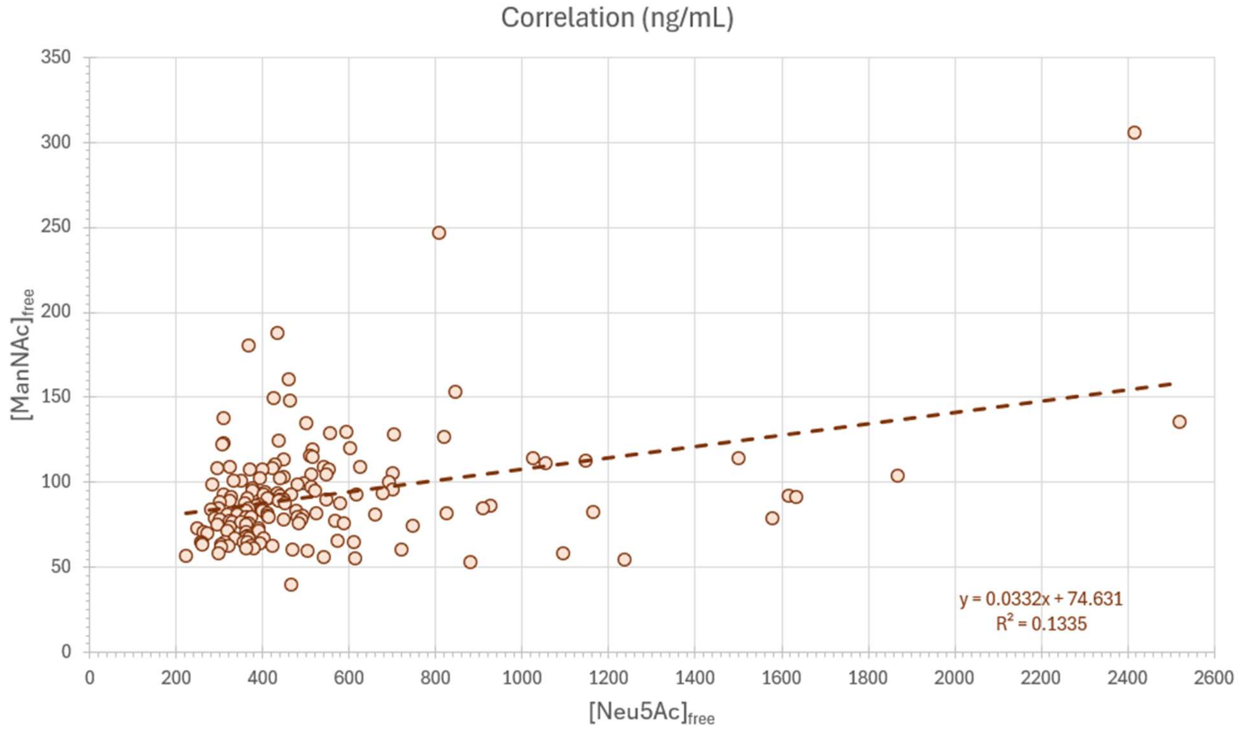

Figure S1. Correlation between free forms of serum Neu5Ac and ManNAc.

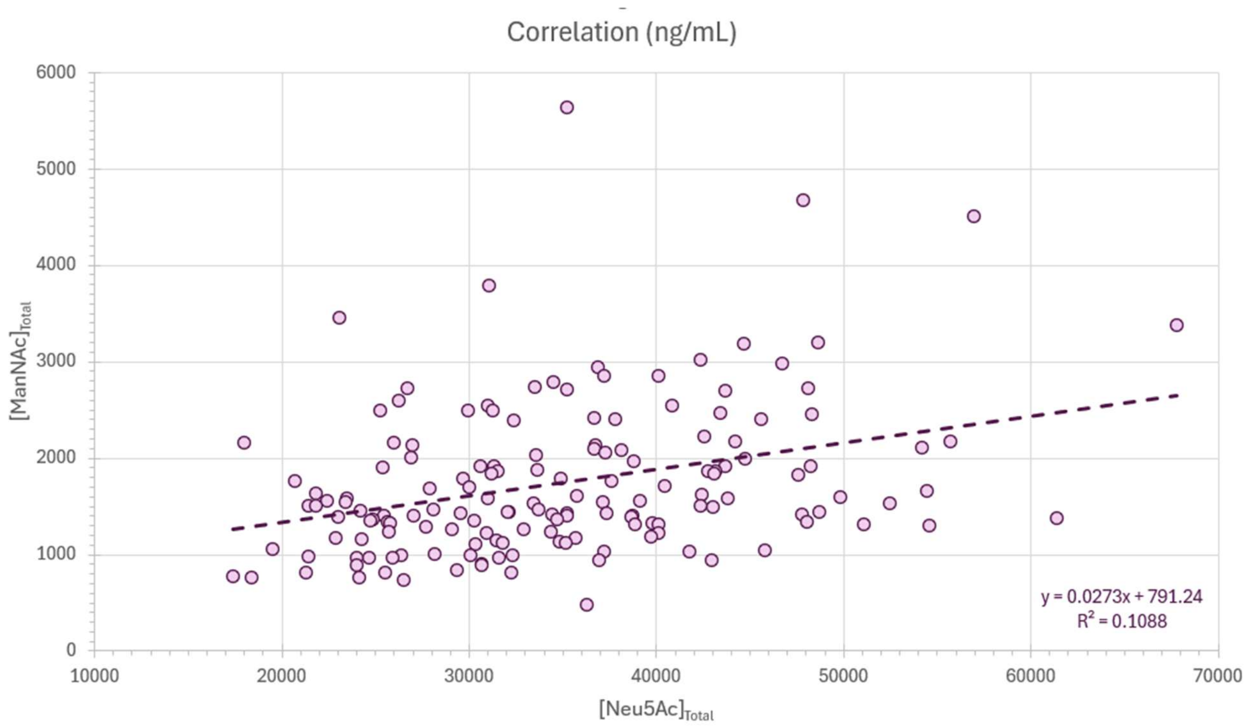

Figure S2. Correlation between serum total Neu5Ac and total ManNAc.

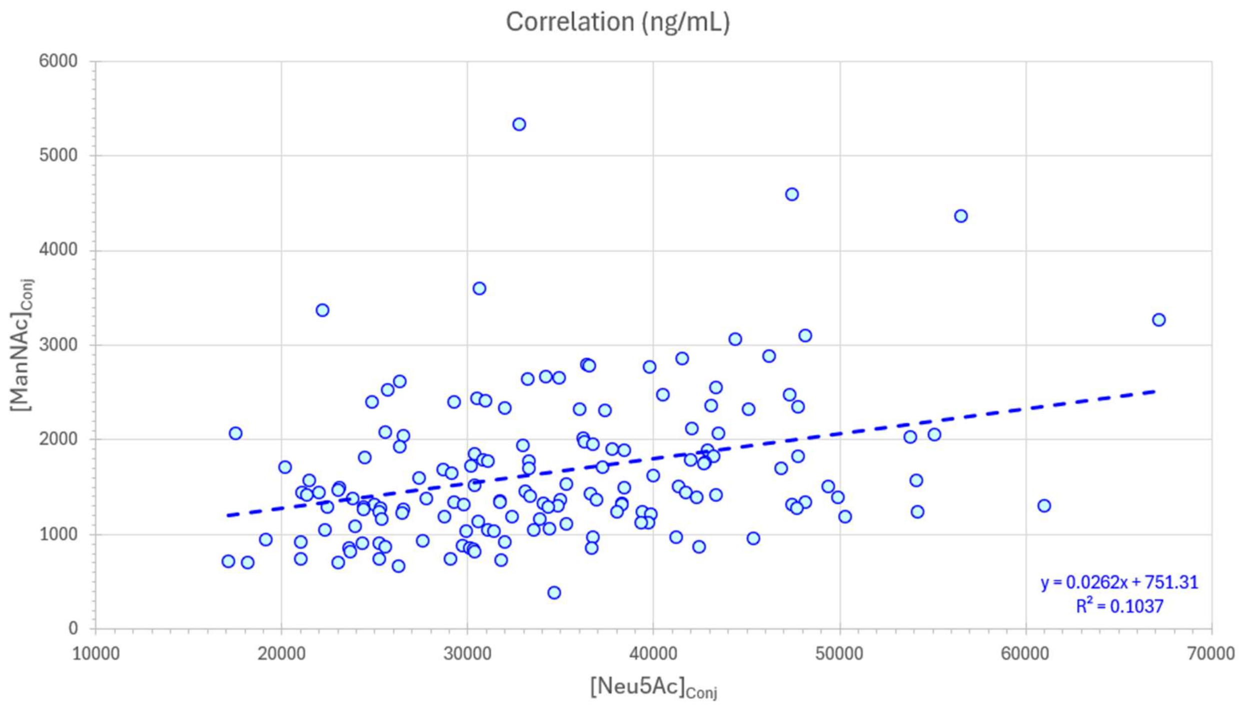

Figure S3. Correlation between conjugated forms of serum Neu5Ac and ManNAc.

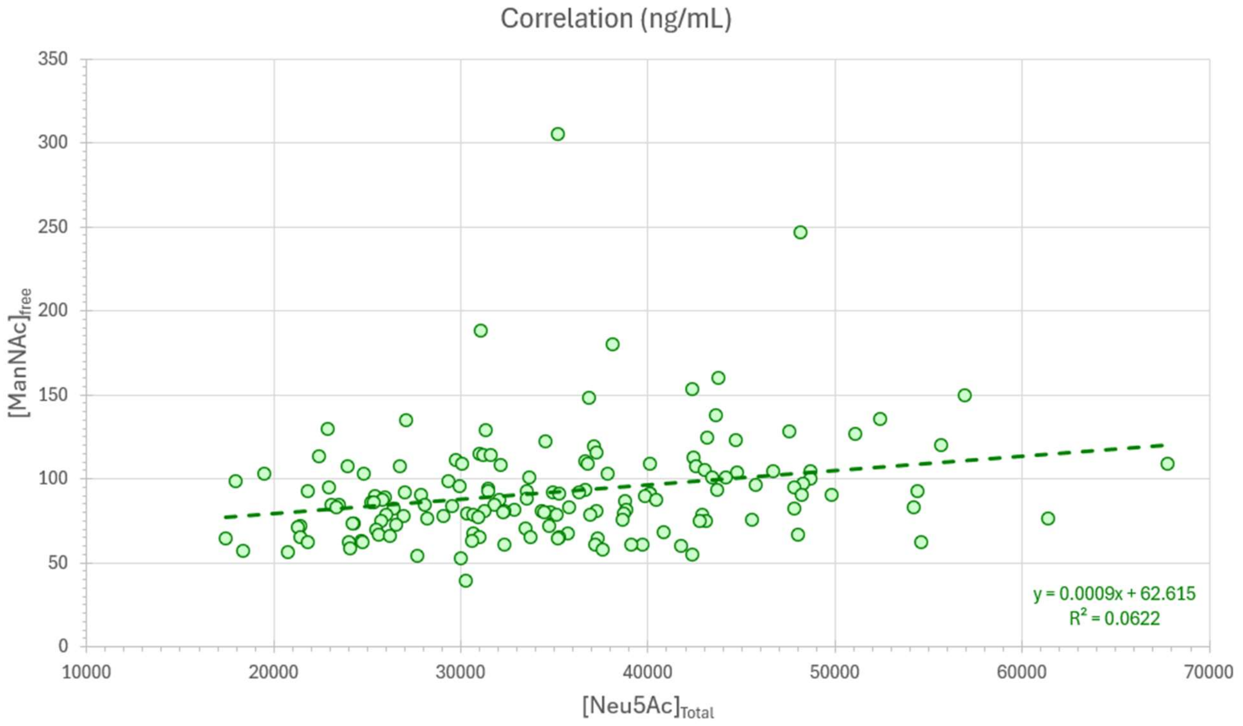

Figure S4. Correlation between the serum total Neu5Ac and free ManNAc.

Table S1. Summary of the linear regression parameters obtained from correlations between Neu5Ac and ManNAc levels measured for 155 participants in the AHS-2. Data is presented based on the format: y = mx + b. SE(m), standard error of the slope m; SE(b), standard error of the intercept b.

Fig.	x	y	m	SE(m)	b	SE(b)	R^2
S1	$Mean[Neu5Ac]_{free}$	$Mean[ManNAc]_{free}$	0.033191	0.006814	74.6309	4.3920	0.133492
S2	$Mean[Neu5Ac]_{Total}$	$Mean[ManNAc]_{Total}$	0.027339	0.006306	791.2388	228.0219	0.108765
S3	$Mean[Neu5Ac]_{Conj}$	$Mean[ManNAc]_{Conj}$	0.026238	0.006216	751.3081	221.4584	0.103711
S4	$Mean[Neu5Ac]_{Total}$	$Mean[ManNAc]_{free}$	0.000855	0.000267	62.6145	9.6714	0.062176
